# Mapping of magnetic resonance imaging’s transverse relaxation time at low signal‐to‐noise ratio using Bloch simulations and principal component analysis image denoising

**DOI:** 10.1002/nbm.4807

**Published:** 2022-08-13

**Authors:** Neta Stern, Dvir Radunsky, Tamar Blumenfeld‐Katzir, Yigal Chechik, Chen Solomon, Noam Ben‐Eliezer

**Affiliations:** ^1^ Department of Biomedical Engineering Tel Aviv University Israel; ^2^ Department of Orthopedics Shamir Medical Center Be'er Ya'akov Israel; ^3^ Sackler Faculty of Medicine Tel Aviv University Tel Aviv Israel; ^4^ Sagol School of Neuroscience Tel Aviv University Israel; ^5^ Center for Advanced Imaging Innovation and Research (CAI^2^R) New York University School of Medicine New York New York USA

**Keywords:** denoising, MRI image denoising, quantitative MRI, T_2_ mapping, T_2_ relaxation

## Abstract

High‐resolution mapping of magnetic resonance imaging (MRI)’s transverse relaxation time (T_2_) can benefit many clinical applications by offering improved anatomic details, enhancing the ability to probe tissues’ microarchitecture, and facilitating the identification of early pathology. Increasing spatial resolutions, however, decreases data's signal‐to‐noise ratio (SNR), particularly at clinical scan times. This impairs imaging quality, and the accuracy of subsequent radiological interpretation. Recently, principal component analysis (PCA) was employed for denoising diffusion‐weighted MR images and was shown to be effective for improving parameter estimation in multiexponential relaxometry. This study combines the Marchenko–Pastur PCA (MP‐PCA) signal model with the echo modulation curve (EMC) algorithm for denoising multiecho spin‐echo (MESE) MRI data and improving the precision of EMC‐generated single T_2_ relaxation maps. The denoising technique was validated on simulations, phantom scans, and in vivo brain and knee data. MESE scans were performed on a 3‐T Siemens scanner. The acquired images were denoised using the MP‐PCA algorithm and were then provided as input for the EMC T_2_‐fitting algorithm. Quantitative analysis of the denoising quality included comparing the standard deviation and coefficient of variation of T_2_ values, along with gold standard SNR estimation of the phantom scans. The presented denoising technique shows an increase in T_2_ maps' precision and SNR, while successfully preserving the morphological features of the tissue. Employing MP‐PCA denoising as a preprocessing step decreases the noise‐related variability of T_2_ maps produced by the EMC algorithm and thus increases their precision. The proposed method can be useful for a wide range of clinical applications by facilitating earlier detection of pathologies and improving the accuracy of patients' follow‐up.

## Graphical abstract

This study evaluates the performance of the Marchenko–Pastur principal component analysis (MP‐PCA) algorithm for denoising T_2_ relaxation maps. The technique was successfully validated on phantom and in vivo scans, showing an increase in T_2_ maps' precision while preserving anatomical features. The ensuing ability for high‐resolution mapping of T_2_ relaxation times can benefit a wide range of clinical applications and enhance the ability for early detection of subtle tissue abnormalities.

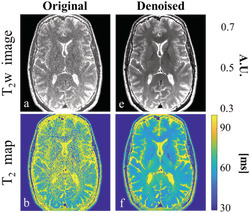

**Abbreviations used**
BM3D: block matching and 3D filtering
CV: coefficient of variation
EMC: echo modulation curve
ETL: echo‐train‐length
MP‐PCA: Marchenko–Pastur PCA
MESE: multiecho spin‐echo
NLM: nonlocal means
PCA: principal component analysis
ROI: region of interest
SNR: signal‐to‐noise ratio
SD: standard deviation

## 1 INTRODUCTION

Recent techniques for quantitative mapping of magnetic resonance imaging (MRI)’s transverse relaxation time (T_2_) offer high precision, improved reproducibility, and have the potential to reveal subtle pathological changes that are not visually apparent on standard contrast‐weighted images.^1^, ^2^ Numerous studies have demonstrated the utility of T_2_ mapping for various clinical applications, including detection and diagnosis of brain ischemic stroke,^3^ cancerous lesions,^4^, ^5^, ^6^ multiple sclerosis,^7^, ^8^ Parkinsonism,^9^ myocardial edema,^1^ and evaluation of cartilage damage.^10^

High‐resolution mapping of T_2_ relaxation times can benefit all of these applications by offering improved anatomic details, enhancing the ability to assess tissue architecture and identify early pathology.^11^ Further to that, some applications necessitate the use of high in‐plane resolution and thin slices for accurate analysis, for example, when scanning the pituitary gland, the internal auditory canal, the articular cartilage, the spine, or the cortical layers of the brain.^12^, ^13^, ^14^, ^15^ Increasing the spatial resolution, however, decreases the data's signal‐to‐noise ratio (SNR). This impairs the quality of the resulting images and the accuracy of the ensuing radiological interpretation.

Various methods have been developed to increase SNR by signal denoising. Bilateral filtering (BF) makes use of a weighted sum of pixel intensities in a localized neighborhood, with the weight calculation taking into account both intensity differences and spatial distance.^16^ Nonlocal means (NLM), a method which can be viewed as a generalization of BF, is based on a weighted average of all of the pixels in the image, and has the advantage of using region comparison rather than pixel comparison. It is, however, highly dependent on the algorithm parameters, and maintains a trade‐off between the precision of measured values and the spatial resolution of the corresponding maps.^17^, ^18^ Block matching and 3D filtering (BM3D) is another approach, based on applying a 3D transformation on a group of similar 2D image fragments (blocks), which are then returned to their original positions after being transformed.^19^, ^20^ BM3D is known to be highly effective, yet more complex, less flexible, and slower than other basic methods, while still suffering from residual artifacts when applied on highly noisy data.^21^ Using low rank modeling for the purpose of noise reduction in MR images is another well studied approach.^22^, ^23^, ^24^, ^25^, ^26^, ^27^, ^28^, ^29^ Recently, principal component analysis (PCA) was employed for the denoising of diffusion‐weighted MR images,^30^, ^31^, ^32^, ^33^, ^34^ and was shown to be effective for improving parameter estimation in multiexponential relaxometry,^18^ quantitative mapping of the spinal cord,^35^ and fMRI language mapping in patients with brain tumors.^36^ In all of the mentioned studies, the redundancy of the scanned data allows the use of low‐rank representation of the signal. Such representation can be extracted using PCA, by identifying and removing the principal components that are associated with thermal noise. In this work we evaluated the utility of PCA‐based complex image denoising for improving the quality of T_2_ mapping from rapid multiecho spin‐echo (MESE) data. The extraction of accurate T_2_ values from MESE signals is not trivial to begin with, due to the inherent and unavoidable deviation of the signal from the theoretical exponential decay pattern.^37^ This deviation is caused by imperfect refocusing slice profiles and inhomogeneities of the transmit magnetic field (B_1_^+^). An effective tool that overcomes these deviations is the echo modulation curve (EMC) algorithm, offering highly accurate mapping of single T_2_ values in vivo.^7^, ^38^, ^39^ This technique is based on fitting experimental MESE signals to a precalculated dictionary of theoretical decay curves generated using Bloch simulations that are tailored to the MRI scanner and scan settings being used.^40^ Specifically, this is achieved by incorporating the exact RF pulse shapes, gradient events, and B_1_^+^ inaccuracies into the dictionary generation process, thereby producing accurate, precise, and reproducible T_2_ values.^7^ Notwithstanding its performance, the EMC algorithm may still suffer from reduced precision when operating at low SNR, particularly for fast relaxing T_2_ components.

To address this limitation, in this study we employed the Marchenko–Pastur PCA (MP‐PCA) algorithm to denoise MESE relaxometry data and incorporated it as an additional preprocessing step to the EMC algorithm. The performance of the combined platform is demonstrated using numerical simulations, physical phantoms, and on in vivo brain and knee data.

## 2 METHODS

### 2.1 Numerical simulation

**FIGURE 1:**
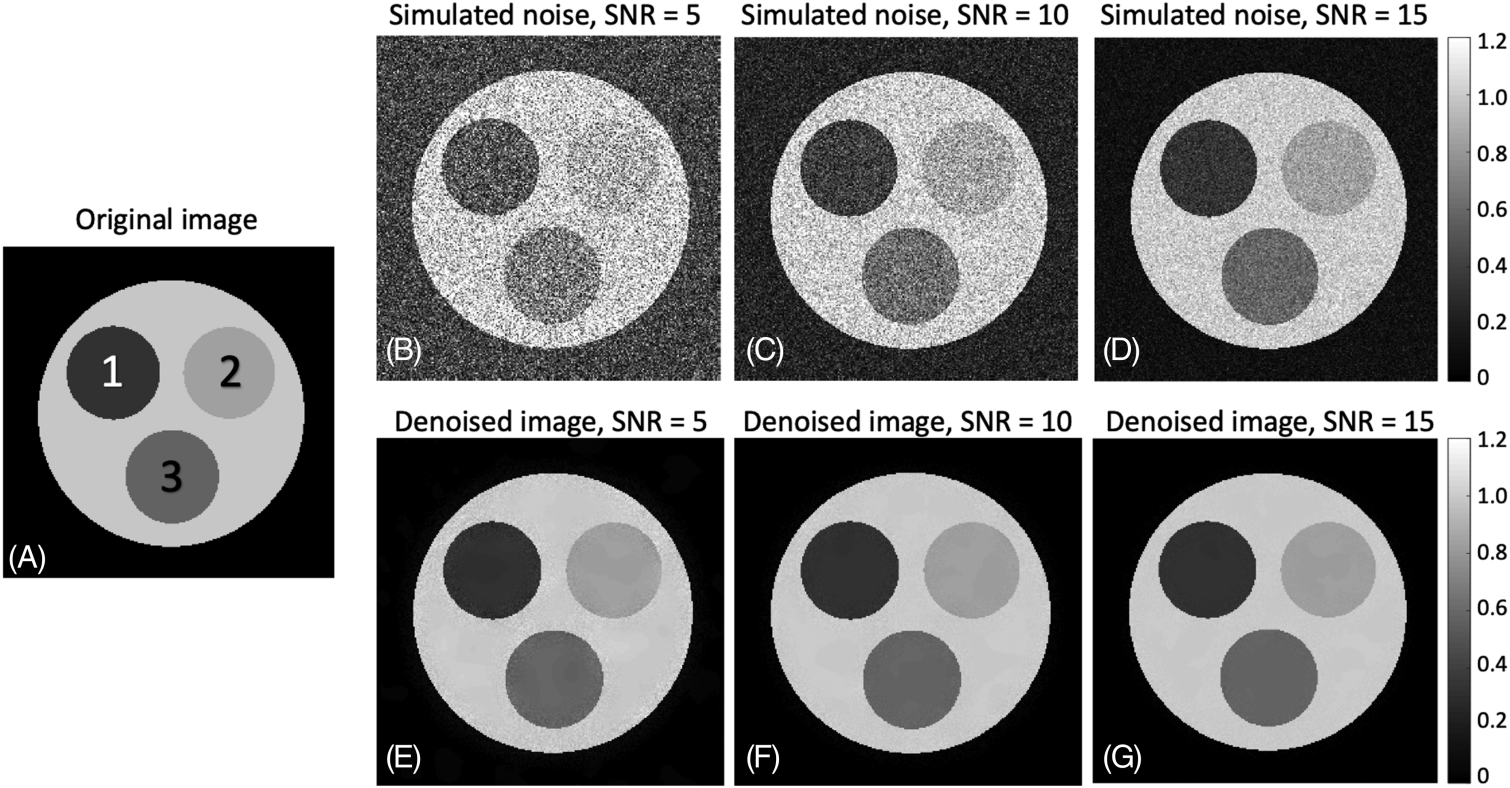
Numerical phantom simulations showing T_2_‐weighted images predenoising and postdenoising (fifth echo time, echo spacing of 10 ms). (A) An image of the numerical phantom showing three internal compartments: #1 and #2 contain single T_2_ components (30 and 100 ms, respectively), while compartment #3 contains a mixture of the two values (30 and 100 ms). (B)‐(D) Images with simulated noise (SNR = 5, 10, 15). (E)‐(G) Denoised images. SNR, signal‐to‐noise ratio

Numerical phantom simulations were developed and executed using MATLAB R2018b. Figure 1A shows the numerical phantom consisting of three internal compartments. Two contain single T_2_ components of 30 and 100 ms, and the third compartment contains a bi‐component signal generated by mixing the above two components with equal fractions.

To test the algorithm performance under different settings, four configurations were used in the simulations, using TE = 10,15 ms and echo‐train‐length (ETL) = 15, 30. A second set of simulations was performed for TE = 10 ms across different ETL values of 5, 10, 15, 20, 25, and 30. The decay curves used in the simulations were generated using the EMC algorithm described below.^40^ After reconstructing images for all echo times, white Gaussian noise was added to the complex images using a method described by Does et al.^18^ Images with simulated noise were then denoised using the MP‐PCA algorithm.

### 2.2 MRI scans

All scans were performed on a 3‐T whole‐body MRI scanner (Siemens Healthineers Inc.). Phantom scans were performed on the T_2_ array of the ISMRM/NIST phantom (HPD Inc.). The phantom was scanned 10 times using a standard MESE protocol, using a range of scan parameters consisting of slice thickness = 1 and 2 mm and matrix sizes = 100 x 100, 192 x 192, 256 x 256, 380 x 380, and 512 x 512, with total scan times of 5 min 3 s, 9 min 39 s, 12 min 51 s, 19 min 3 s, and 25 min 39 s. The remaining scan parameters were: N_Echoes_ = 25; TE/TR = 13/3000 ms; echo spacing = 13 ms; refocusing flip angle = 180°; N_slices_ = 1; field of view (FOV) = 160 mm x 160 mm; and acquisition BW = 200 Hz/Px. No undersampling was applied.

In vivo scans were performed after obtaining informed consent. Brain scans were approved by the institutional review board (IRB) committee of the Sheba Medical Center (protocol number: 3933–17‐SMC). Knee scans were approved by the IRB committee of the Shamir Medical Center (protocol number: 0098–18‐ASF). Brain imaging was performed for two healthy volunteers. Data were acquired using a 64‐ and a 20‐channel head coil and by using the same standard MESE sequence. Scan parameters for the first volunteer were: slice thickness = 3 mm, matrix size = 216 x 180, FOV = 216 mm x 180 mm, TE/TR = 10/3000 ms, echo spacing = 10 ms, N_Echoes_ = 15, N_slices_ = 15, acquisition BW = 210 Hz/Px, and total scan time: 9 min 3 s. No undersampling was applied. Scan parameters for the second volunteer were: slice thickness = 1 mm, matrix size = 324 x 300, FOV = 192 mm x 178 mm, TE/TR = 12/2500 ms, echo spacing = 12 ms, refocusing flip angle = 180°, N_Echoes_ = 20, N_slices_ = 10, GeneRalized Autocalibrating Partial Parallel Acquisition (GRAPPA) acceleration factor = 2, acquisition BW = 205 Hz/Px, and total scan time: 6 min 48 s. Knee scans were performed on a single healthy volunteer. Scan parameters were: slice thickness = 1.5 mm, matrix size = 448 x 280, FOV = 192 mm x 120 mm, TE/TR = 12.1/2500 ms, echo spacing = 12.1 ms, refocusing flip angle = 180°, N_Echoes_ = 15, N_slices_ = 10, GRAPPA acceleration factor = 2, BW = 199 Hz/Px, and total scan time: 6 min 23 s. Knee images and T_2_ maps were inspected by an orthopedist with 8 years of experience, to assess the potential improvement in the diagnostic quality of denoised data.

### 2.3 Image denoising using MP‐PCA

This section delineates the procedure for denoising T_2_ relaxation data, which were acquired using rapid MESE imaging protocols. The implementation presented herein is based an existing denoising algorithm published recently by Does et al. for multicomponent signal analysis.^18^, ^30^ A three‐dimensional complex image matrix is collected from each receiver coil with the following dimensions (*N*_RO_, *N*_PE_, ETL), denoting the number of frequency‐encoding points (RO), phase‐encoding points (PE), and ETL time points. Data are denoised for each individual coil and each slice separately, and the ensuing images are ultimately combined per slice using standard sum of squares.^41^ The denoising procedure is performed using a sliding window, with the window size scaled proportionally to matrix size (e.g., a 15 x 15 x 15 window for a matrix size of 324 x 300 and ETL = 15).

Let *N*_*v*_ be the number of voxels in each window and *N*_*E*_ be the number of echoes. A two‐dimensional matrix $A\in {\mathbb{C}}^{N_{E}\times N_{v}}$ is created, for which each column represents the time‐dependent signal in a single voxel, and the rows represent different voxel values per each echo time. The matrix A is normalized into $\bar{A\;}=A-\bar{x}1_{1\;x\;N_{v}}$, where $\bar{x}\in {\mathbb{C}}^{N_{E}\;x\;1}\;$ is the mean signal across the set of *N*_*v*_ voxels. Using singular value decomposition,^42^ $\bar{A}$ is transformed into $\mathrm{US}V^{T}$, where the columns of U are the eigenvectors of ${\bar{A}\bar{A}}^{T}$, the columns of V are the eigenvectors of ${\bar{A}}^{T}\bar{A}$, and S is a diagonal matrix containing the singular values of $\bar{A}$. Following this, we now define *P* to be the number of principal components associated with the denoised signal. Once *P* is calculated, a new denoised matrix can be retrieved by truncating the USV matrices and adding back the mean signal according to

(1) $$\left[U\left(:,1:P\right)*S\left(1:P,1:P\right)*V{\left(:,1:P\right)}^{T}\right]+\bar{x}1_{1xN_{v}}.$$

The new denoised matrix assigns a new curve to each voxel in the window. Because each voxel appears in several overlapping windows (e.g., 25 windows for a 5 x 5 x 15 window), the MP‐PCA algorithm produces several denoised curves for each voxel. The mean of the denoised curves is then assigned to the voxel. In the special case where *P* = 0, the new denoised matrix is equal to $\bar{x}1_{1xN_{v}}$, meaning that no signal is detected at that voxel and all its columns are all equal to $\bar{x}$. This is common among background voxels, for which the received signal contains only noise.

Previous studies demonstrated different approaches for selecting *P*.^22^, ^29^ The approach selected in this study is based on the distribution of the eigenvalues of the data covariance matrix. Let *M* = min (*N*_*v*_, *N*_*E*_) – *P* be the total number of the lowest eigenvalues of $\bar{A}\bar{A}$ ^**T**^ that are associated with noise. Assuming the noise level is constant among all columns of $\bar{A}$, these *M* eigenvalues can be described by the Marchenko–Pastur distribution.^18^, ^30^, ^43^ This leads to a simplified approach for finding *P*, as presented by Veraart et al.,^30^ stating that the selected *P* should be equal to the minimum value of $\bar{P}$, for which

(2) $$\frac{\lambda_{\bar{P}+1}-\lambda_{M}}{4\sqrt{\left(M-\bar{P}\right)∕N_{v}}}<\frac{\sum_{i=\bar{P}+1}^{M}\lambda_{i}}{\left(M-\bar{P}\right)\;}.$$

Once denoising is applied on the series of MESE images, T_2_ maps are generated using the EMC algorithm.^40^ For the sake of brevity, below we describe the gist of this algorithm. As its basis, the EMC algorithm uses a full Bloch simulation of the prospective MESE MRI protocol to generate theoretical T_2_ decay curves (i.e., EMCs) for each predefined T_2_ and transmit field (B_1_^+^) inhomogeneity value. This process is then repeated for the range of T_2_ values expected to appear in the tissue, in our case T_2_ = 10–600 (ms), and B_1_^+^ levels of 70%–130% (where 100% denotes a perfectly homogeneous value). The result of this process is a dictionary of EMCs, each associated with a unique [T_2_,B_1_^+^] value pair. Once the experimental EMC is acquired for a given voxel it is denoised, normalized to the first time point (i.e., all EMCs start at unity), and then matched to the set of dictionary entries by calculating the L2 norm of the difference between the denoised EMC and the dictionary EMCs. The entry with the minimal norm determines the [T_2_,B_1_^+^] value pair for that voxel. Repeating this process for all image voxels then produces the final T_2_ and B_1_^+^ maps.

To exclude echoes with low SNR from the calculation, the L2 norm of the difference is not calculated across all echoes but excludes the tail of the signal. Selection of the number of echoes to include in the fitting process is performed by examining the signal variations starting at the last four time points and gradually moving backwards to the first echo. The inverse of the coefficient of variation (CV), that is, the mean divided by the standard deviation (SD), will, in this case, be initially high, containing only noisy data points, and gradually decreasing as the mean signal increases. The point at which this value drops below a predefined threshold is determined as the last echo containing meaningful data. An illustration of this process is provided in Figure **S1**.

### 2.4 Estimation of SNR predenoising and postdenoising

Predenoising and postdenoising brain images were examined by a neuroradiologist with 11 years of experience, to estimate changes in the quality of the images and to ascertain whether morphological features are preserved. Predenoising and postdenoising knee images were similarly examined by an orthopedist with 8 years of experience.

In addition to estimating the visual improvement of the denoised images, we also estimated the SNR enhancement in the phantom and brain images. Phantom SNR was calculated for six spheres, representing a physiological range of brain T_2_ values (according to manufacturer‐reported values). Regions of interest (ROIs) representing the signal intensity were marked within the internal region of each sphere using the magnitude images from the first echo. A second set of ROIs was marked on the images' background to assess the noise level. The SNR was then calculated by dividing the mean value within each sphere by the SD of noise in the background ROIs. The SD of the normally distributed complex noise was calculated as the mean value of the magnitude image within these regions divided by $\sqrt{\pi /2}.$ ^44^ Another possible method for calculating the SNR was to divide the mean value within each ROI by the SD of the within the same ROI; however, this method was not used due to Gibbs artifacts affecting the SD inside the spheres (see Figure S2). A second measure for the increase in SNR postdenoising was performed by calculating the mean, SD, and CV of T_2_ values within each sphere. Because each sphere contains a homogeneous chemical solution, any variation in its T_2_ values can be attributed to noise.

SNR in brain images was assessed for eight brain segments manually delineated on the first echo image. The ROIs were the genu of corpus callosum, left/right caudate nucleus, left/right putamen, left/right thalamus, and the splenium of corpus callosum. The mean, SD, and CV of T_2_ values were calculated within each ROI.

Three ROIs were marked on T_2_ maps of the knee: ROI #1, patellar cartilage; ROI #2, anterior zone of femoral knee cartilage; and ROI #3, posterior zone of femoral knee cartilage. To demonstrate the decrease in T_2_ values between the original and denoised maps, T_2_ mean and SD were calculated for these regions, and compared between the two maps.

## 3 RESULTS

Figure 1 shows images generated using numerical phantom simulations. The three compartments are marked on Figure 1A: compartments #1 and #2 contain a single T_2_ component of 30 and 100 ms, respectively, while compartment #3 contains a mixture of the two. Figure 1B‐D show the simulated images after random noise addition (SNR = 5, 10, and 11), and Figure 1E‐G show the same images after applying MP‐PCA image denoising with a window size of 15 x 15 x ETL. Effective denoising with edge preservation are clearly seen when comparing the upper and lower rows.

To test the performance of MP‐PCA denoising as a function of the number of echoes, simulations were also performed for different ETLs. Table 1 shows the T_2_ mean and SD of the single‐component compartments #1 and #2 of the numerical phantom. Simulations used echo spacings of 10 ms, and a denoising window size of 15 x 15 x ETL. High variability and SDs are observed for T_2_ values predenoising, whereas denoised values exhibit highly consistent mean T_2_ regardless of the ETL, with significantly reduced SDs.

**TABLE 1:** Mean and standard deviation (SD) of T_2_ values for a pair of single T_2_ compartments in a numerical phantom predenoising and postdenoising (SNR = 10). Simulations were performed using echo‐spacing (ms) and across different ETL values to test the performance of MP‐PCA denoising as a function of the number of echoes. The window size used for denoising was 15 x 15 x ETL. The use of PCA denoising reduces the variability of mean values and the corresponding SDs, yielding consistent T_2_ values regardless of the ETL being used

T_2_ (ms)	ETL	T_2_ values			
		Predenoising		Postdenoising	
		Mean (ms)	SD (ms)	Mean (ms)	SD (ms)
30	5	51.49	87.04	29.37	0.63
	10	33.08	10.94	29.76	0.43
	15	33.54	8.41	30.00	0.00
	20	35.31	7.90	29.99	0.12
	25	37.01	9.00	29.96	0.19
	30	38.47	9.22	29.93	0.25
100	5	157.45	146.97	100.31	1.73
	10	154.33	144.47	96.57	3.43
	15	119.74	77.92	99.07	0.89
	20	110.24	44.33	99.48	0.53
	25	106.40	21.48	99.56	0.64
	30	105.97	19.02	99.89	0.31

Abbreviations: ETL, echo‐train‐length; MP‐PCA, Marchenko–Pastur principal component analysis; SNR, signal‐to‐noise ratio.

**FIGURE 2:**
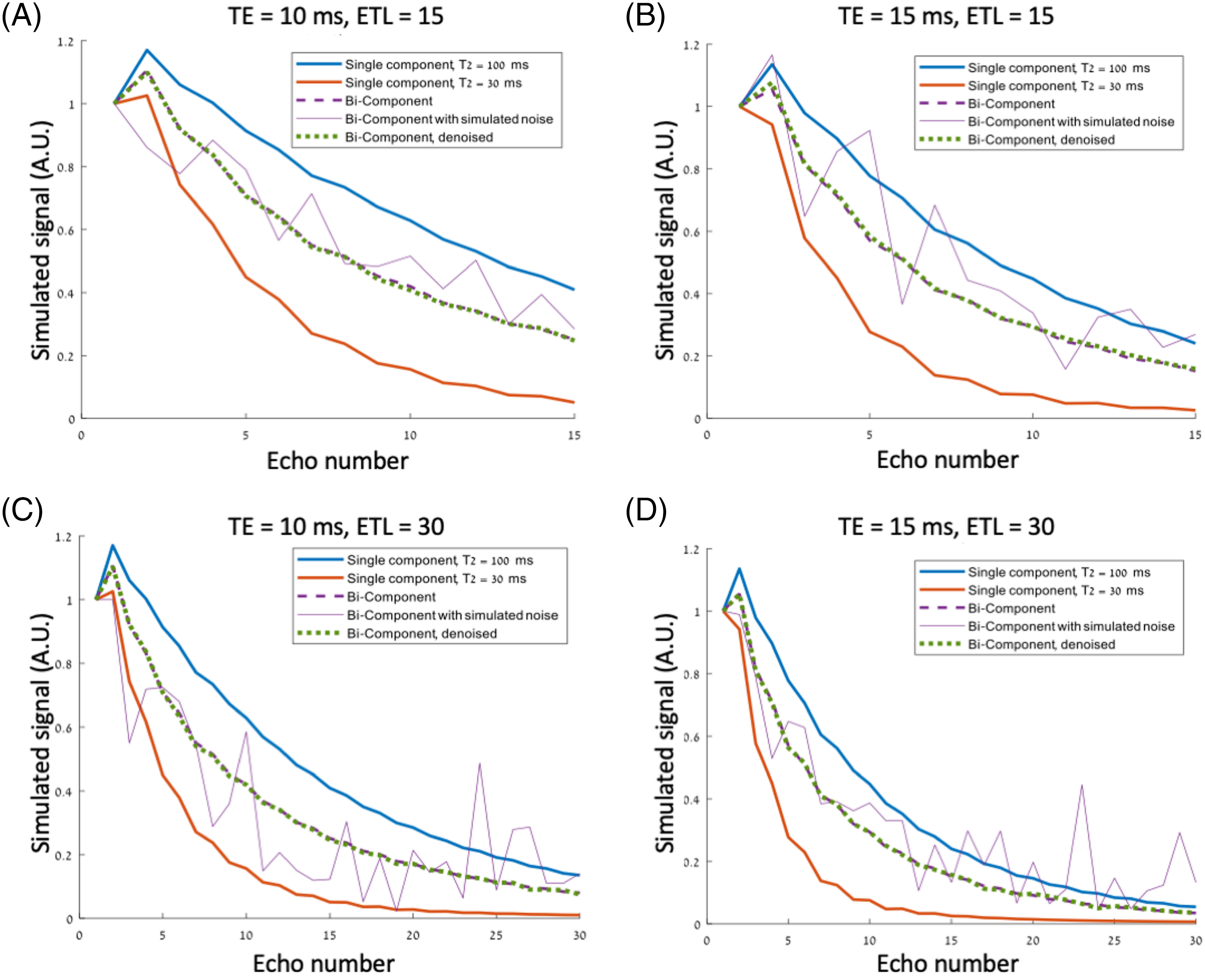
Numerical phantom simulations for a two‐component numerical phantom at two TEs and two ETLs (A‐D). The red and blue curves reflect theoretical single‐component signal decay curves corresponding to T_2_s of 30 and 100 ms, respectively; dashed purple lines show a simulated bi‐component curve reflecting a mixture of the two components; purple lines show the same curve, with noise added (SNR = 10); and the dashed green line shows the curve after applying MP‐PCA 15 × 15 × ETL. ETL, echo‐train‐length; MP‐PCA, Marchenko–Pastur principal component analysis; SNR, signal‐to‐noise ratio

Analysis of the bi‐component signal decay curves is shown in Figure 2 for four different scan settings. As can be seen, the noise‐corrected curves are almost identical to the original curves in comparison with the noisy curves, for all scan settings and across all echo times.

**FIGURE 3:**
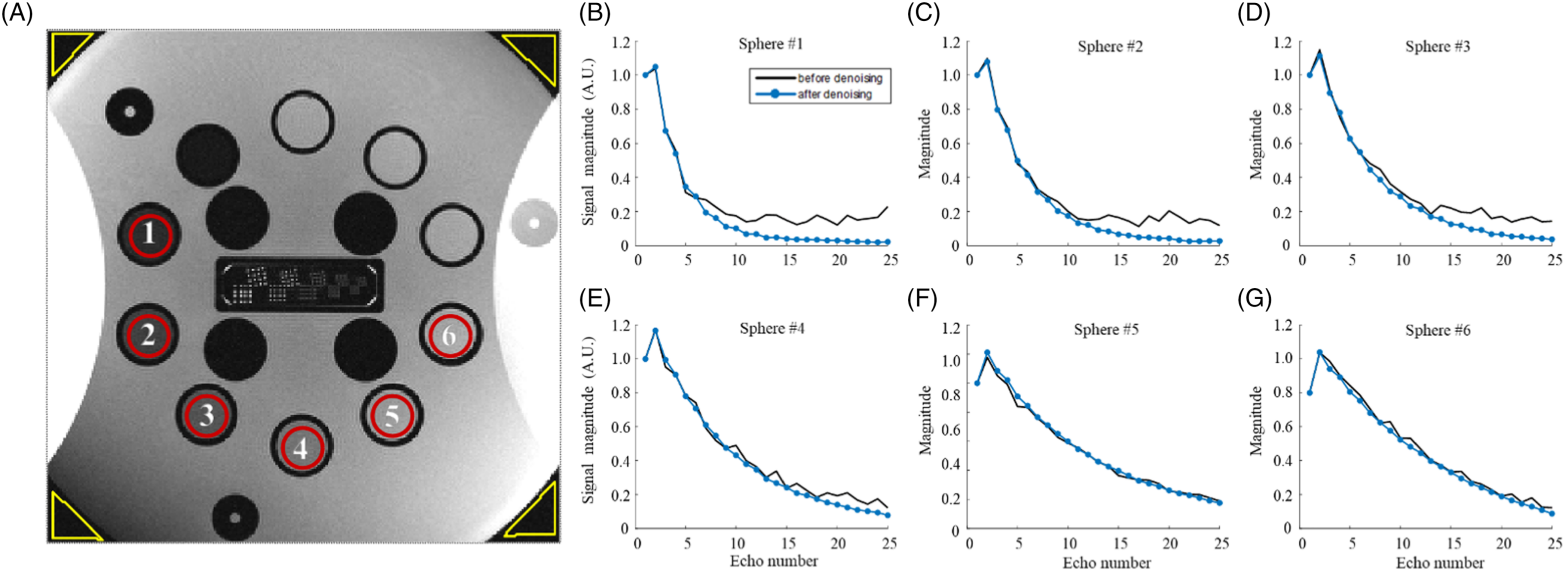
(A) T_2_‐weighted image of the HPD phantom (TE = 78 ms), using slice thickness = 1 mm, matrix size = 512 x 512. ROIs of six spheres were selected for further analysis, having physiological T_2_ values in the range of 30–185 ms. An additional four ROIs in the background (yellow) were selected for the purpose of SNR estimation (see Table 2); (B‐G) Curves from the central voxels of each of the six selected ROIs are shown predenoising and postdenoising. ROI, region of interest; SNR, signal‐to‐noise ratio

Figure 3 shows a T_2_‐weighted image of the HPD phantom (TE = 30 ms), illustrating its internal structure and the six ROIs selected for evaluating the SNR predenoising and postdenoising. To highlight the denoising effect on the underlying T_2_ decay curves, experimental EMCs from the central voxels of each ROI were extracted, and are shown in Figure 3B‐G before and after denoising. As can be seen, signal decay curves for all assayed T_2_ values exhibit reduced variability after the denoising process. Even more pronounced is the almost complete disappearance of a noncentral chi‐distributed noise pattern,^45^ clearly apparent in the tail of the short T_2_ signals (Figure 3A‐C). Table 2 contains the mean, SD, and CV of T_2_ values, alongside the estimated SNR per ROI for scans performed with a 1 mm slice thickness. Scans performed with a slice thickness of 2 mm or above showed little improvement in SNR and are therefore not shown. The values in Table 2 show a significant decrease in SD and CV predenoising and postdenoising, whether using a 5 x 5 x 15 or a 7 x 7 x 15 denoising window. Because each sphere contained a uniform solution, the signal variability within each ROI can be attributed mainly to noise and is thus directly related to the SNR within that ROI. The reduction in SD and CV of T_2_ values indicates the effectiveness of the denoising procedure across the different T_2_ values. A consistent increase in SNR is shown for all six selected spheres and for every resolution tested, once again demonstrating the effectiveness of the denoising procedure. Relative increase in SNR seems to be correlated with image resolution: as the scan resolution increases, the increase in SNR is more prominent. To visually demonstrate the reduction in the spatial variation of T_2_ values after denoising, a close‐up view of the values within spheres #3 and #4 with and without denoising is presented (Figure S3). Further numerical analysis of the noise pattern, removed by the denoising algorithm, is presented in Figure S4.

**TABLE 2:** T_2_ values for the T_2_ array of the HPD phantom across different spatial resolutions. T_2_ mean, standard deviation (SD), coefficient of variation (CV) and SNR per ROI were calculated for six of the HPD phantom spheres. All scans were acquired using a 1 mm slice. SNR was calculated using the magnitude image resulting from the first echo, by dividing the mean value of each ROI with the SD of selected background pixels. A consistent increase in SNR is shown for all the six spheres and for every resolution tested

Sphere no.	Matrix size	No. of voxels in ROI	T_2_ values predenoising				T_2_ values postdenoising (window size = 5 x 5 x 15)				T_2_ values postdenoising (window size = 7 x 7 x 15)			
			Mean (ms)	SD (ms)	CV (%)	SNR	Mean (ms)	SD (ms)	CV (%)	SNR	Mean (ms)	SD (ms)	CV (%)	SNR
1	512 x 512	1243	37.1	10.2	27.6	3.1	30.3	0.5	1.5	20	30.1	0.4	1.2	27.7
2			48.9	11.2	23	3.3	42.4	0.6	1.5	21.5	42.1	0.5	1.3	29.7
3			67.1	13.6	20.3	3.4	58.2	0.8	1.4	22	57.8	0.5	0.9	30.4
4			94	13.8	14.7	3.7	82.1	1.1	1.3	24.5	81.7	0.8	1	33.9
5			136	18.2	13.4	4	119.4	1.5	1.3	27	119	1.2	1	37.4
6			182.3	27.1	14.9	3.9	159	2.8	1.7	26.1	158.7	2.3	1.5	36.1
1	380 x 380	610	34.7	4.1	11.7	4.6	30.2	0.4	1.2	30.9	30	0	0	40.8
2			47	4.2	9	5	42.6	0.5	1.2	33.1	42.3	0.4	1	43.8
3			64.7	6	9.2	5.1	58.5	0.6	1.1	33.8	58.3	0.5	0.9	44.7
4			90.9	6.7	7.3	5.6	83.2	0.7	0.9	37.9	83.1	0.6	0.7	50.1
5			131.5	8.5	6.5	6.2	122.3	1	0.8	41.8	122.2	0.6	0.5	55.3
6			180	14.6	8.1	6	164.7	1.9	1.2	40.2	164.6	1.6	1	53.2
1	256 x 256	311	32.6	1.6	5	8.2	30.1	0.3	1	50.7	30	0.5	1.5	61.8
2			45	1.9	4.1	8.8	42.5	0.5	1.2	54.6	42.4	0.5	1.2	66.4
3			62	2.8	4.6	8.9	58.7	0.6	0.9	55.7	58.6	0.6	1	67.8
4			87.9	3.3	3.8	10	84.3	0.8	1	62.4	84.2	0.8	1	76
5			127.7	3.8	3	11	124.1	0.9	0.7	68.8	124.1	0.7	0.6	83.8
6			174.7	5.9	3.4	10.6	168.3	1.9	1.1	66.4	168.4	1.8	1.1	80.8
1	192 x 192	147	32	1.6	5	12.3	30.2	1	3.2	67	30.2	1.3	4.1	75.1
2			44.3	1.7	3.7	13.2	42.6	0.8	1.9	71.9	42.7	1.1	2.5	80.7
3			60.9	1.9	3	13.5	59	0.6	1.1	73.7	58.9	0.9	1.5	82.6
4			86	2.7	3.1	15.2	84.3	0.9	1.1	83	84.3	1.2	1.4	93.1
5			126.9	3	2.4	16.7	124.3	1.4	1.1	91.3	124.4	1.5	1.2	102.3
6			171.6	4.7	2.7	16.1	168.6	2.5	1.5	87.8	168.7	2.3	1.4	98.4
1	100 x 100	27	30.9	2	6.4	30.4	30.2	2.3	7.6	88.9	30.1	2.3	7.8	88.2
2			43	3.2	7.5	32.6	42.3	3.7	8.8	95.3	42.4	3.7	8.8	94.5
3			59.3	3.2	5.5	33.6	59	3.2	5.4	98.1	59	3.2	5.4	97.2
4			84.7	2	2.3	38	84.3	1.8	2.1	110.9	84.3	1.8	2.1	109.9
5			126.1	5.8	4.6	41.8	126.1	5.9	4.6	122.2	126.1	6.1	4.8	121.1
6			172.9	7.9	4.6	40.4	172.3	7	4.1	118.2	172.1	7.7	4.5	117.1

Abbreviations: ROI, region of interest; SNR, signal‐to‐noise ratio.

**FIGURE 4:**
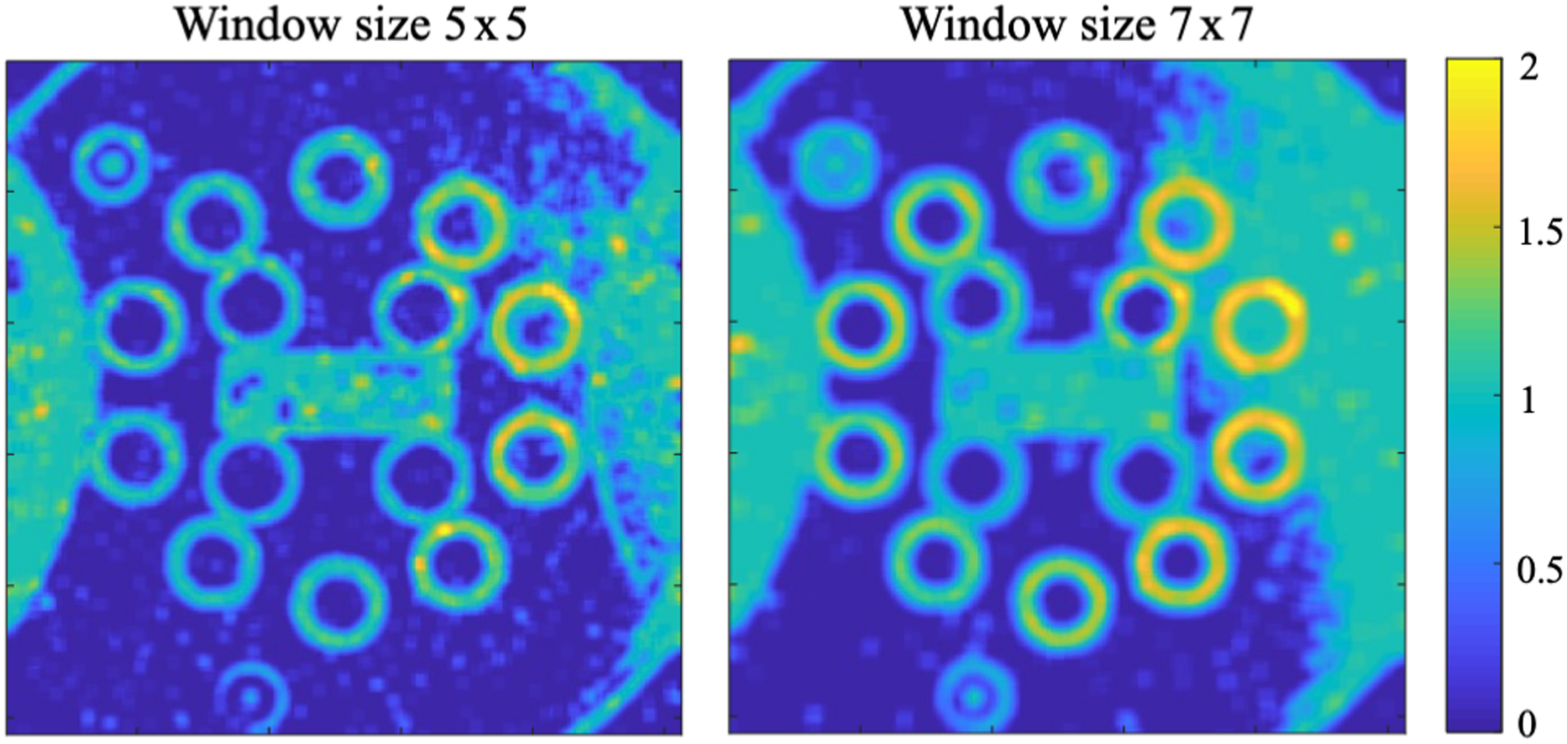
Numerical maps showing the number of principal components found per pixel, divided by the number of windows that include this pixel (“P‐map”). Maps represent data that were extracted from a single receiver channel. The left map was extracted using a 5 x 5 x 15 window and the right map was extracted using a 7 x 7 x 15 window. Original scanning was performed with a 20‐channel receiver coil, producing 20 P‐maps – one per coil. The maps illustrate the advantage of applying PCA denoising on a sliding window: the number of principal components is calculated per window according to its content, allowing to distinguish real edges from background noise. PCA, principal component analysis

The number of principal components, which was used to model the signal in each denoising window, directly correlates to the amount of structural information within that window. To validate the denoising algorithm's ability to retain the structural features of the imaged object, in Figure 4 we show parametric maps containing the number of principal components used to produce the denoised image in each voxel for window sizes 5 x 5 x 15 and 7 x 7 x 15. The number of principal components was further normalized by the number of moving windows that contained that specific voxel (termed the “P‐map”), extracted from the data of a single channel. Scans were performed using a 20‐channel receiver coil, resulting in 20 separate P‐maps. These maps illustrate the advantage of applying PCA denoising on a sliding window: the number of principal components is calculated per window according to its content level, allowing to effectively distinguish background from edges and preserve the morphological features of the object.

**FIGURE 5:**
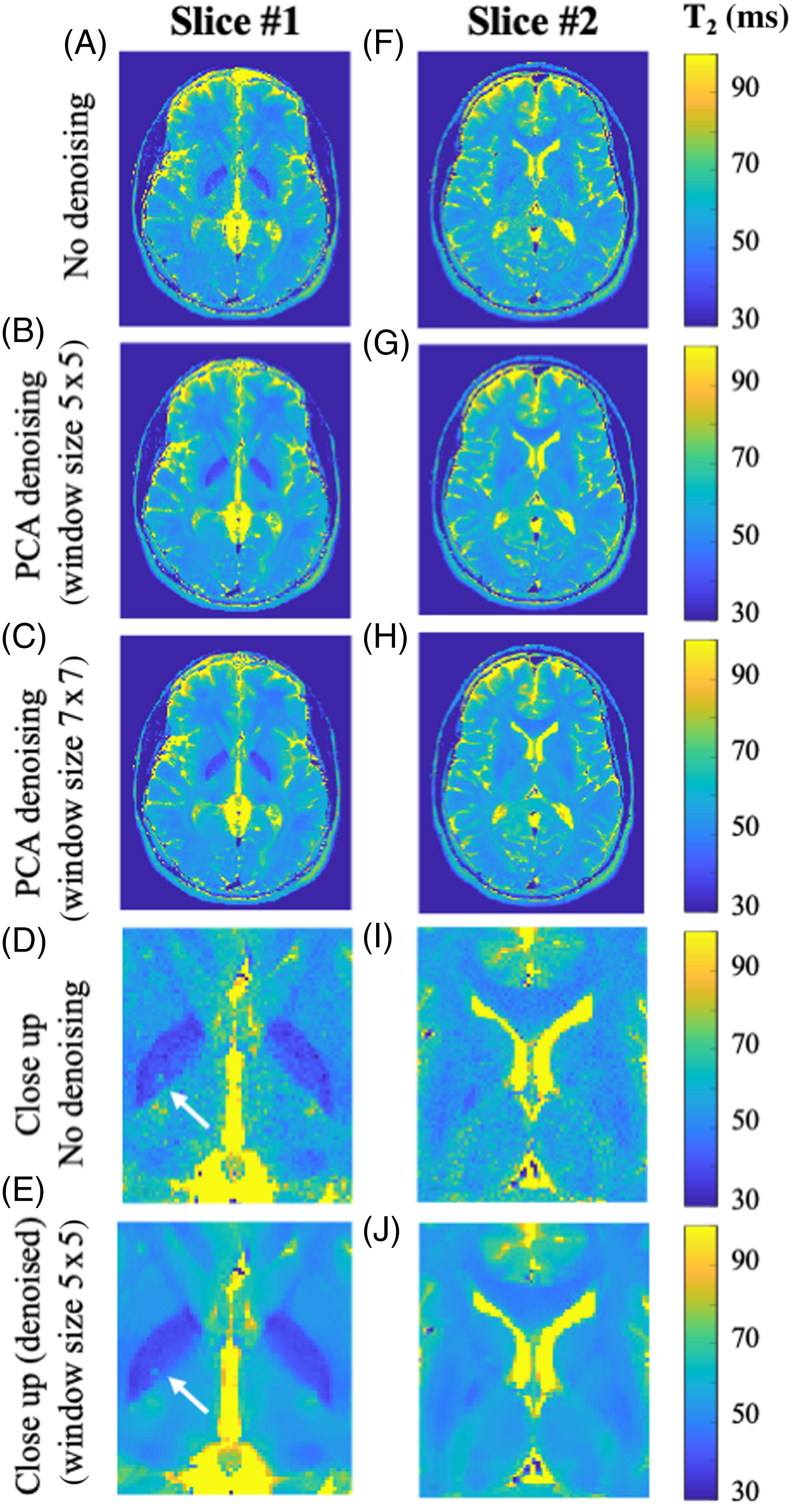
T_2_ maps from the first in vivo brain scan. Each column contains T_2_ maps extracted for a different slice. The first row (A,F) contains EMC‐fitted maps without denoising. The second and third rows (B‐C, G‐H) contain maps that were extracted after applying MP‐PCA image denoising with 5 x 5 x 15 and 7 x 7 x 15 windows, respectively. The fourth and fifth rows (D‐E, I‐J) show close‐up views of two zoomed ROIs predenoising and postdenoising, demonstrating the algorithm's ability to preserve structural features. EMC, echo modulation curve; MP‐PCA, Marchenko–Pastur principal component analysis; ROI, region of interest

**FIGURE 6:**
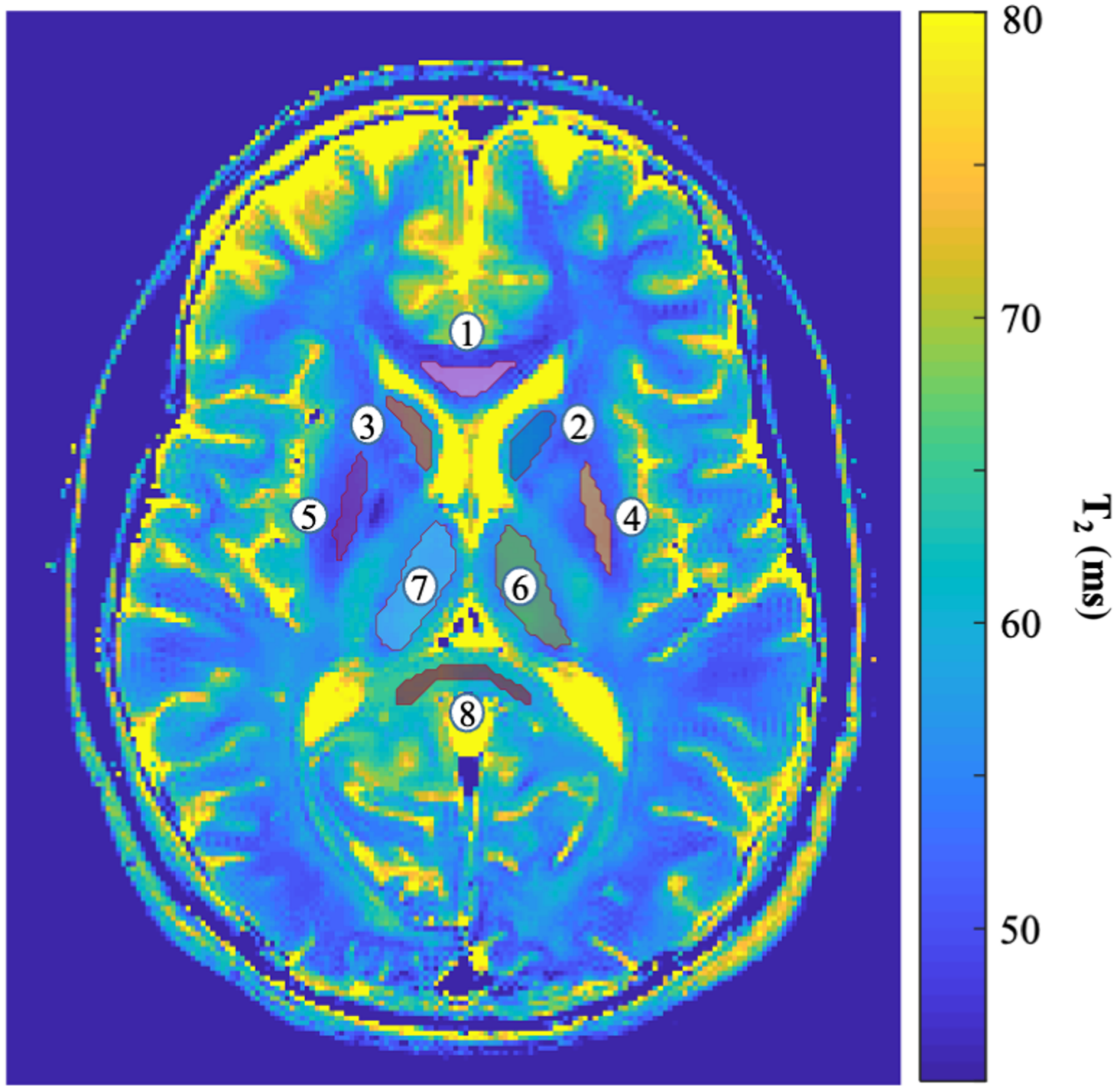
Selected ROIs from slice #2, overlaid on a denoised T_2_ map (window size 5 x 5 x 15). ROIs were extracted using FreeSurfer software. The selected regions are: (1) Genu of corpus callosum; (2) Left caudate; (3) Right caudate; (4) Left Putamen; (5) Right Putamen; (6) Left Thalamus; (7) Right Thalamus; and (8) Splenium of corpus callosum. ROI, region of interest

**FIGURE 7:**
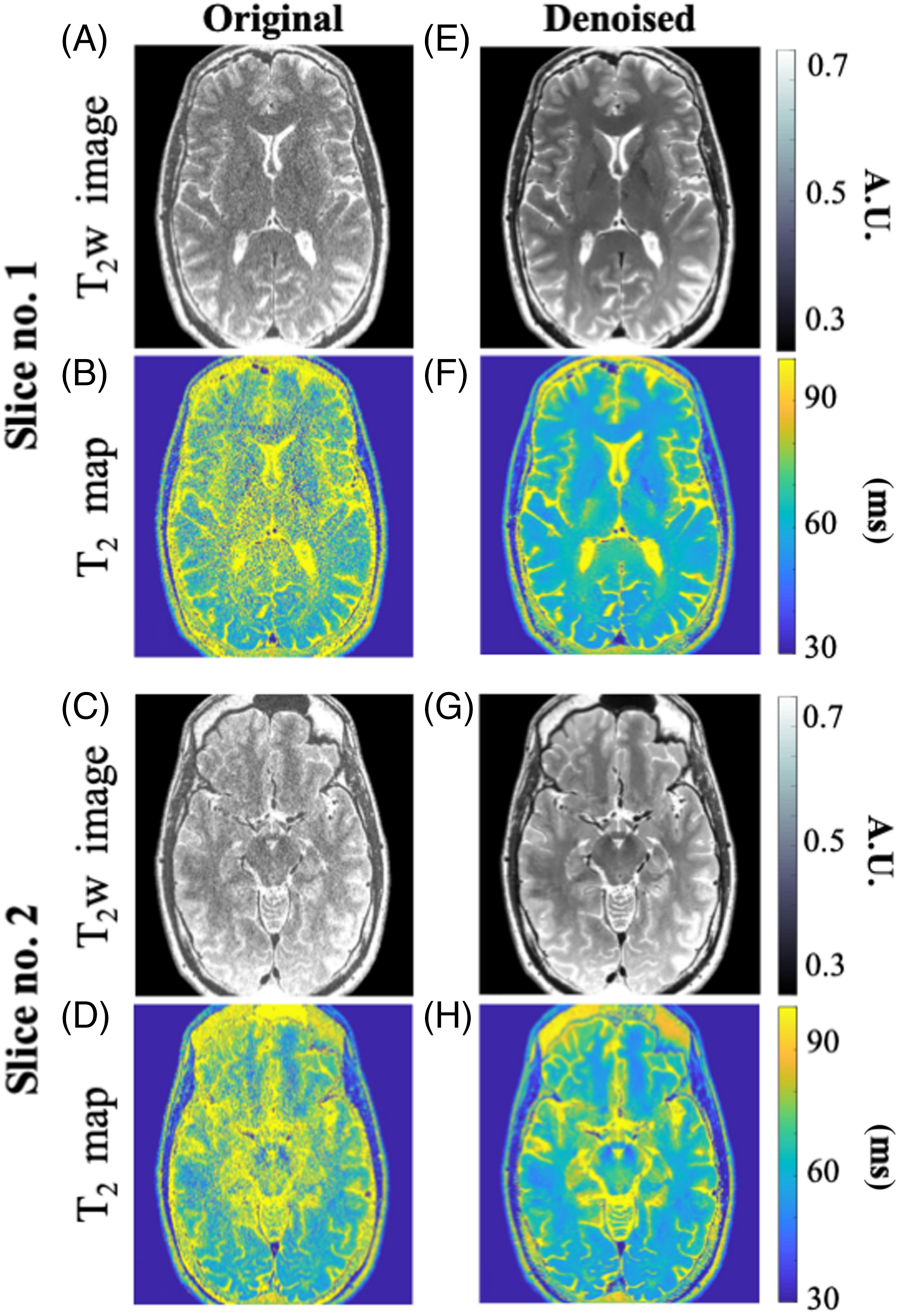
T_2_‐weighted images (A,C,E,G) and quantitative T_2_ maps (B,D,F,H) for two slices from the second (high‐resolution) brain scan. Left/right columns show the predenoising/postdenoising images and T_2_ maps (window size 15 x 15 x 20)

**FIGURE 8:**
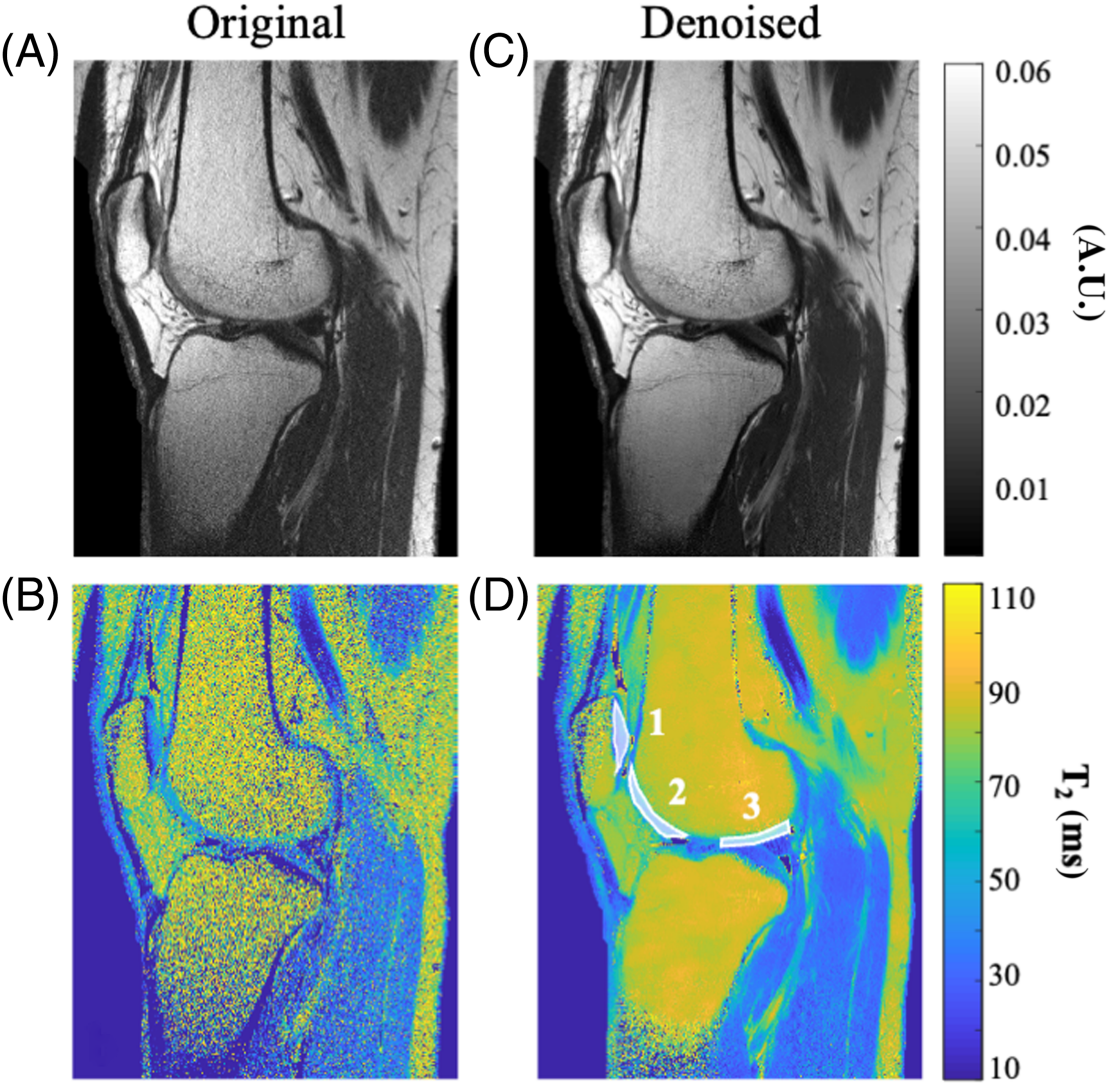
T_2_‐weighted images (A,C) and T_2_ maps (B,D) of a selected slice acquired using a high‐resolution in vivo knee scan (matrix size = 448 x 280, FOV = 192 x 120 mm^2^). The left column shows the original images and T_2_ maps, and the right column shows the corresponding images and T_2_ maps after MP‐PCA image denoising (window size 20 x 10 x 15). The three ROIs shown in (D) were marked for the purpose of calculating and comparing T_2_ values predenoising and postdenoising. FOV, field of view; MP‐PCA, Marchenko–Pastur principal component analysis; ROI, region of interest

Images from the brain and knee scans were examined by a neuroradiologist and an orthopedist, who both reported a clear improvement in the images' quality, and no loss of morphological features. Results from the in vivo scans are presented in Figures 5, 6, 7, 8. Figure 5 shows results from the first of two in vivo brain scans. Each column contains T_2_ maps of a different slice. The first row presents T_2_ maps that were calculated based on the original data, while the second and third rows contain maps calculated after applying PCA complex image denoising using a 5 x 5 x 15 and a 7 x 7 x 15 window, respectively. Noise reduction is apparent for both slices, with no apparent difference between the two window sizes. The last two rows present close‐up views of the T_2_ maps of both slices. No apparent loss of spatial resolution can be seen in the denoised maps (Figure 5B,C,G,H). This is lucidly demonstrated by examining the perivascular space, indicated by white arrows in Figure 5D,E, and appearing in both the original images as well as in the denoised ones. This example demonstrates the ability of the denoising algorithm to preserve real anatomical features, even as small as four voxels in size, and to differentiate them from background noises. This was further demonstrated when repeating the same MESE scans several times (Figure S5), demonstrating how real anatomical features are preserved by the algorithm, while noise‐related patterns are removed. Table 3 delineates the mean T_2_ values for the series of ROIs shown in Figure 6, segmented on slice #2 from Figure 5. Original T_2_ values are shown, alongside values calculated from denoised maps using 5 x 5 x 15 and 7 x 7 x 15 window sizes. For both window sizes, the inclusion of the PCA‐denoising step reduces SD and CV. A consistent decrease in T_2_ mean value is also apparent postdenoising because of the reduction in noncentral chi‐distributed noise, which elevates the tail of the T_2_ decay curve.

**TABLE 3:** In vivo T_2_ values of selected ROIs predenoising and postdenoising. Left to right: T_2_ mean, SD, and CV generated from the original images; from PCA‐denoised images using a 5 x 5 x 15 window; and from PCA‐denoised images using a 7 x 7 x 15 window. The PCA‐denoising step reduces the SD and CV of values. A small decrease in T_2_ mean value is also apparent postdenoising due to correction of the bias resulting from noncentral chi‐distributed noise. The selected ROIs and their indices are presented in Figure 6

	T_2_ values predenoising			T_2_ values postdenoising (window size = 5 x 5 x15)			T_2_ values postdenoising (window size = 7 x 7 x 15)			
	Mean (ms)	SD (ms)	CV (%)	Mean (ms)	SD (ms)	CV (%)	Mean (ms)	SD (ms)	CV (%)	
**Genu of corpus callosum (ROI1)**	50.6	2.9	5.7	49.6	1.3	2.6	49.3	1.3	2.5
**Left caudate (ROI2)**	57.9	4.6	8	56.4	2.5	4.5	56.4	2.4	4.3
**Right caudate (ROI3)**	58.4	3.7	6.3	57.4	2	3.5	57.4	2	3.4
**Left putamen (ROI4)**	50.7	2.9	5.8	50.7	1.2	2.3	50.5	1.5	3
**Right putamen (ROI5)**	51.2	2.8	5.5	50.8	1.5	2.9	50.6	1.6	3.2
**Left thalamus (ROI6)**	56	4.2	7.4	55.7	1.9	3.3	55.4	1.9	3.5
**Right thalamus (ROI7)**	56.9	4.6	8.1	56.1	1.7	3.1	56	1.8	3.2
**Splenium of corpus callosum (ROI8)**	64.2	6.1	9.6	62.4	2.5	4	62.5	2.5	4

Abbreviations: CV, coefficient of variation; PCA, principal component analysis; ROI, region of interest; SD, standard deviation.

Figure 7 shows T_2_‐weighted images and the corresponding T_2_ maps for two different slices, acquired using a high‐resolution scan (voxel size 0.6 x 0.6 x 1 mm^3^). Figure 7A‐D show the original images and T_2_ maps, whereas Figure 7E‐H show the denoised images and maps. Because of the high resolution of these data, a large window size of 15 x 15 x 20 voxels was used for denoising. A significant increase in SNR can be seen in the denoised images and maps, with no apparent loss of structural features. A second set of high‐resolution scans of the knee is provided in Figure 8, showing original and denoised T_2_‐weighted images and T_2_ maps of a single sagittal slice. We note that in the T_2_ map originating from the nondenoised images, some central voxels in the bones were assigned with zero values due to failure of the fitting algorithm in low‐SNR areas; thus the values in these voxels do not reflect real T_2_ values. Visual inspection of these images and maps shows a consistent increase in SNR and preservation of features, similar to our findings in brain and phantom data. Analysis for all regions shows a decrease in T_2_ mean and SD after denoising. Calculated T_2_ values for the patellar cartilage (ROI #1) were 41.3 ± 14 and 35.5 ± 7 ms in the original and denoised T_2_ maps, respectively. Calculated T_2_ values in the anterior zone of femoral knee cartilage (ROI #2) were 54.1 ± 18.3 and 48.9 ± 13.7 ms in the original and denoised T_2_ maps, respectively. Calculated T_2_ values in the posterior zone of femoral knee cartilage (ROI #3) were 62.7 ± 24.2 and 50.7 ± 7.9 ms in the original and denoised T_2_ maps, respectively.

A visual demonstration of feature preservation in the in vivo scans is provided in Figure S7, presenting zoomed‐in views of selected regions in the brain and knee scans.

## 4 DISCUSSION

In this study we utilized the MP‐PCA algorithm to improve the SNR of quantitative T_2_ maps produced by the EMC algorithm.^40^ The technique was successfully validated on phantoms and in vivo, showing an increase of the maximal achievable resolution. Two of the in vivo scans were performed using GRAPPA undersampling, both for the purpose of reducing scan time, and to validate the MP‐PCA processing on undersampled datasets.

A clear removal of noise‐related bias of T_2_ values was seen in the phantom results. Similar results were obtained for the brains, with a more modest change in the first brain scan. This can be explained by the difference in slice thickness, which was 1 mm for the phantom scan compared with 3 mm in the in vivo brain scan. As a result, SNR levels in the brain images were initially higher, with reduced noise‐related bias of the magnitude images used for T_2_ mapping.^44^

The anatomy of the cartilage and surrounding soft‐tissues of the knee was reported to be more accurate to radiologic inspection after denosiging. This indicates the potential of the combined platform to improve the diagnosis of musculoskeletal pathologies such as menisci tears, soft tissue lesions, and cartilage defects. Another example is osteochondral lesions, which have been commonly diagnosed in orthopedic surgery in recent years, increasing the need for more accurate imaging capabilities. Indeed, a clearer and sharper contour of the cartilage is observed in the denoised T_2_ maps. Due to the sharpness of cartilage matrix, the physis can be imaged more accurately and the posterior root of lateral meniscus is better delineated, which may help in identifying related pathologies.

Quantitative analysis of the suggested technique included comparing the SD and CV of T_2_ values for different baseline T_2_ values, along with direct SNR estimation in the phantom scans. Our interpretation of the in vivo results relies on the assumption that the reduced SD in the selected ROIs results from noise reduction, and not from an undesired spatial smoothing. We make this nontrivial assumption based on the phantom results, which showed a clear preservation of features. This was manifested in the automatic selection of higher values of *P* in regions containing edges, and selection of lower values of *P* in background regions or areas of uniform signal intensity (Figure 4). The ability of the MP‐PCA complex image denoising to retain structural features indeed stems from its ability to selectively change the number of principal components (*P*) for each window based on its information content. As can be seen in Figure 4, for example, the value of *P* increases when the algorithm is applied on areas that contain edges. Intuitively, the time‐dependent data in these windows can be interpreted as redundant matrices, for which most variability is spread across one to three principal axes. For windows containing background pixels or windows located in a uniform region, the value of *P* will be close or equal to zero. To obtain a visual illustration of the noise reduction, the difference between predenoising and postdenoising was calculated for a sample pair of images, and is shown in Figure S6. The difference map shown reflects a nonuniform spatial distribution of the relative noise being removed with some of the anatomical structures visible. Although we would theoretically expect the noise maps to exhibit no structural information, this arises from the fact that the algorithm chooses a different number of principal components in each window. For instance, the center of the anatomy is farthest away from the receive coils, thus having the lowest SNR. As can be seen in the relative difference map, this is the area that is most affected by the denoising.

Previous studies examined other algorithms for eigenvalue thresholding and selection of *P*, for example, the NORDIC method.^32^ MP‐PCA was selected because it was previously employed on MESE data for the purpose of multiexponential T_2_ analysis (by Does et al.^18^), while NORDIC was applied on diffusion MRI (dMRI). Moreover, applying NORDIC requires the use of noise images, which we do not have access to. Still, similarities exist between dMRI and MESE data, suggesting that NORDIC might also be effective for denoising MESE data. Evaluation and analysis of this method are left for future research.

The MP‐PCA denoising algorithm relies on the assumption that the noise eigenvalues are MP‐distributed. MR images, however, are typically presented in magnitude format having noncentral chi‐distributed noise, which may not sustain this assumption. The use of magnitude images may therefore lead to selecting a wrong number of principal components. This is indeed corroborated by the results from Does et al., who tested the algorithm for denoising multi‐T_2_‐component data.^18^ In that study, it was found that the denoising efficacy was reduced for magnitude images compared with complex images. The preliminary tests we performed on magnitude images of phantoms have validated this finding (results not shown). We therefore applied the algorithm on the complex data extracted separately for each receive channel.

Examination of Table 2 shows a consistent decrease in the mean T_2_ values of the HPD phantom after denoising. This bias is increased as spatial resolution increases (and SNR decreases), an effect which is also demonstrated in the in vivo measurements. This can be explained by the presence of noncentral chi‐distributed noise in the magnitude images. As we progress along the echo train, the images' SNR decrease, while the noise elevates the tail of the signal, leading to an overestimation of the T_2_ values. This T_2_ bias is resolved by the denoising process, and also by truncating time points that are dominated mostly by noise. This was implemented using a procedure that selects the appropriate number of echoes per pixel, as described in the Methods section and in Figure **S1**. We further note that denoised T_2_ values across different spatial resolutions exhibit significantly reduced variability thanks to the removal of noncentral chi‐distributed noise (Table 2).

Although the simulated phantom included a two‐component compartment, a single T_2_‐fitting algorithm was used in this study. While the denoising algorithm may have a different effect on each of the underlying components, a rigorous analysis of denoising multicomponent signals is beyond the scope of this study and is left for future work.

Implementing MP‐PCA denoising on clinical scanners has several limitations. First, the extraction of complex images in row data format is not automatic. Second, image denoising is performed separately for each receiver channel, leading to extensive computation time and memory usage. The issue of computation time was handled in this study by parallelizing the denoising and reconstruction of each channel across different CPU cores. Still, applying the denoising algorithm on, for example, a 380 x 380 slice using a 5 x 5 x 15 window size took 2 min 32 s, raising the need for more efficient reconstruction procedures to enable real‐time applications. An important aspect is that the MP‐PCA algorithm is designed to remove thermal noise only, as opposed to noise that originates from other sources such as undersampling artifacts.^30^ Moreover, thermal noise variance is spread across all principal components, meaning that signal‐associated principal components also contain residual noise. Noise is consequently not completely removed by omitting noise‐associated principal components. From a theoretical point of view, assuming the noise level is constant and uncorrelated within the local neighborhood and across MESE measurements, applying the algorithm on regions characterized by *P* > 0 is expected to provide an SNR increase of $\sqrt{\min\left(N_{E},N_{V}\right)/P}$, which, for example, translates to a 3.87‐fold increase in SNR using common values of N_E_ = 15, N_V_ = 25, and *P* = 1.^30^ Increasing the size of the sliding window, however, introduces additional noise patterns and higher spatial variability. The window size should therefore be carefully considered, even in cases where $\;N_{E}\;\text{is less than}\;N_{V}$. In addition, GRAPPA might introduce spatial correlation in the noise pattern, which can potentially decrease the denoising quality. The denoising, however, was still effective when applied on the second brain scan, which was performed using GRAPPA (see Figure 7). Lastly, the two‐region method used herein to calculate SNR, while highly common, relies on the assumption of spatially uniform noise distribution, which is not necessarily met when using parallel imaging techniques.^46^ More accurate SNR estimation will require a large number of repetitions, entailing significantly longer scan times and involving other factors affecting interscan variations. We thus found it appropriate to use the two‐region method despite its shortcomings, as it still correlates to the true images' SNR, and thereby to the effectiveness of the presented denoising technique.

The window size for the MP‐PCA image denoising process was selected empirically per scan. After testing different window sizes for different in vivo scans, we found that applying MP‐PCA denoising using a relatively small window size resulted in “cloud‐like” image artifacts, while using a large window resulted in grainy images and low denoising quality. This is demonstrated on denoised in vivo images in Figure S8. Further discussion on the considerations involved with window size selection is described in Veraart et al.^30^

In this work we used the sum of squares method for combining denoised images from different coil channels. Adaptive combination^47^ is another technique for coil combination that introduces spatial effects due to its reliance on spatially varying coil sensitivities. We avoided this to ensure as clear as possible estimation of the denoising quality, factoring out the effects of external elements such as coil sensitivity profiles. Further research using adaptive combination or other coil combination methods has the potential to improve SNR levels even further.

## 5 CONCLUSIONS

In this study we implemented MP‐PCA image denoising to improve the precision of the EMC T_2_‐mapping algorithm. The combined pipeline was successfully validated on both phantom and in vivo scans, leading to an improvement in the T_2_ maps’ precision while preserving anatomical features. This new capability will enable precise quantification of T_2_ values, even at high spatial resolutions. The proposed method can benefit a wide range of clinical applications by facilitating earlier detection of pathologies and providing more accurate follow‐up of patients.

## Supporting information


**Figure S1:** Example for the steps used by the MP‐PCA denoising algorithm, in order to exclude noisy times points from the T_2_ fitting process. **(a)** Original pre‐denoising image, corresponding to the second TE of a MESE acquisition. **(b)** The original signal decay curve of a single voxel, marked as a red dot in (a). **(c)** The signal decay curve after a 5‐point moving average. For each group of values starting from the last echo, the inverse of the coefficient of variation (mean / SD) is calculated. The value of the inverse of the coefficient is initially high, containing only noisy data‐points, and gradually decreases as the mean signal and SD increase. The point, at which this value drops below a threshold = 6.0, is chosen as the last echo containing meaningful signal.
**Figure S2:** Gibbs ringing artifacts shown on the first echo‐time images of two MESE scans. Both scans were performed using the same FOV (160x160 mm^2^) and using matrix sizes of 256x256 (left) and 100x100 (right). As can be seen, both images, and particularly the low resolution one, are affected by Gibbs ringing artifacts, preventing reliable estimation of the noise pattern (i.e., the standard deviation of the signal) within each homogeneous sphere.
**Figure S3: Left column:** Close up view of the T_2_ maps constructed from original images for spheres 3 and 4 (top and bottom rows). **Right column:** Close up view of the T_2_ maps constructed from denoised images for the same spheres. Color axis was adjusted separately to the relevant T_2_ range of each sphere. A clear decrease in T_2_ values' variability can be seen post‐denoising.
**Figure S4:** Mean of the removed noise across echoes, evaluated for internal ROIs of spheres #2 (~42 ms) and #5 (~120 ms) of the phantom. Noise was calculated by subtracting the denoised images from the original images. Analysis of sphere #2 revealed an increase in noise mean, while analysis for sphere #5 showed a less significant trend.
**Figure S5:** T_2_ maps resulting from three identical MESE acquisitions of the same anatomy. **(a‐c)** T_2_ maps generated from the original images; **(e‐g)** T_2_ maps generated from denoised images; **(d)** and **(h)** show the averages of (a‐c) and (e‐g), respectively. The feature marked by the red rectangle was identified by an expert as an anatomical feature (a perivascular space); as can be seen in (e‐g), this feature was preserved in the denoising process for all repetitions. The region marked by the white rectangle contains several voxels with exceptionally high T_2_ values. Comparing the spatial variance within this region in (a‐c), it is likely that the variance of the presented T_2_ values is this region are affected by noise. The apparent noise removal in this region can be examined when comparing panels (a‐c) to (e‐f).
**Figure S6:** Spatial distribution pattern of removed noise **(a)** The original image acquired at the 8th echo of an in vivo brain MESE scan. **(b)** The corresponding denoised image (window size 5x5x15). **(c)** Difference between the left and the middle images.
**Figure S7:** Demonstration of feature preservation by comparing zoom‐in views into four selected images. **(a‐d)** First brain scan; **(e‐f)** Second brain scan; **(g‐h)** Knee scan. Left column: original T_2_ maps; Right column: denoised T_2_ maps.
**Figure S8:** Examination of denoising results for brain imaging with different window sizes. Each row contains images of a different slice from the second high‐resolution brain scan mentioned in the paper. Denoising with a small window size results with “cloudiness” effects, while using a large window results in grainy images.
