# Supplementary material for: Mapping of magnetic resonance imaging’s transverse relaxation time at low signal‐to‐noise ratio using Bloch simulations and principal component analysis image denoising
Source: NMR Biomed. 2022 Aug 13;35(12):e4807. doi: 10.1002/nbm.4807 (PMC9787782; doi:10.1002/nbm.4807)
Supplement: Supplementary file 1 — Figure S1: Example for the steps used by the MP‐PCA denoising algorithm, in order to exclude noisy times points from the T2 fitting process. (a) Original pre‐denoising image, corresponding to the second TE of a MESE acquisition. (b) The original signal decay curve of a single voxel, marked as a red dot in (a). (c) The signal decay curve after a 5‐point moving average. For each group of values starting from the last echo, the inverse of the coefficient of variation (mean / SD) is calculated. The value of the inverse of the coefficient is initially high, containing only noisy data‐points, and gradually decreases as the mean signal and SD increase. The point, at which this value drops below a threshold = 6.0, is chosen as the last echo containing meaningful signal. Figure S2: Gibbs ringing artifacts shown on the first echo‐time images of two MESE scans. Both scans were performed using the same FOV (160x160 mm2) and using matrix sizes of 256x256 (left) and 100x100 (right). As can be seen, both images, and particularly the low resolution one, are affected by Gibbs ringing artifacts, preventing reliable estimation of the noise pattern (i.e., the standard deviation of the signal) within each homogeneous sphere. Figure S3: Left column: Close up view of the T2 maps constructed from original images for spheres 3 and 4 (top and bottom rows). Right column: Close up view of the T2 maps constructed from denoised images for the same spheres. Color axis was adjusted separately to the relevant T2 range of each sphere. A clear decrease in T2 values' variability can be seen post‐denoising. Figure S4: Mean of the removed noise across echoes, evaluated for internal ROIs of spheres #2 (~42 ms) and #5 (~120 ms) of the phantom. Noise was calculated by subtracting the denoised images from the original images. Analysis of sphere #2 revealed an increase in noise mean, while analysis for sphere #5 showed a less significant trend. Figure S5: T2 maps resulting from three identical MESE acquisiti [file NBM-35-e4807-s001.docx]

**Mapping of MRI’s T_2_ relaxation time at low SNR using Bloch simulations and principal component analysis image denoising**

# Online supplementary materials

## I. Echo truncation method used by the EMC algorithm

The matching process of the EMC algorithm is based on finding the simulated echo-modulation-curve that is closest to the acquired experimental curve in terms of the L2 norm of the difference between the two. In order to exclude echoes with low SNR from the calculation, this L2 norm is not calculated across all echoes, and excludes echoes that belong to the tail of the signal. Selecting the number of echoes to include in the matching process is done by smoothing the curve, then examining the signal variations starting at the *last* four time points and gradually moving backwards to the first echo. The inverse of the coefficient-of-variation, i.e., mean divided by the standard deviation (SD), will, in this case, be initially high, containing only noisy data-points, and gradually decrease as the mean signal increases. The point, at which this value drops below a predefined threshold is set as the last echo that contains meaningful data.


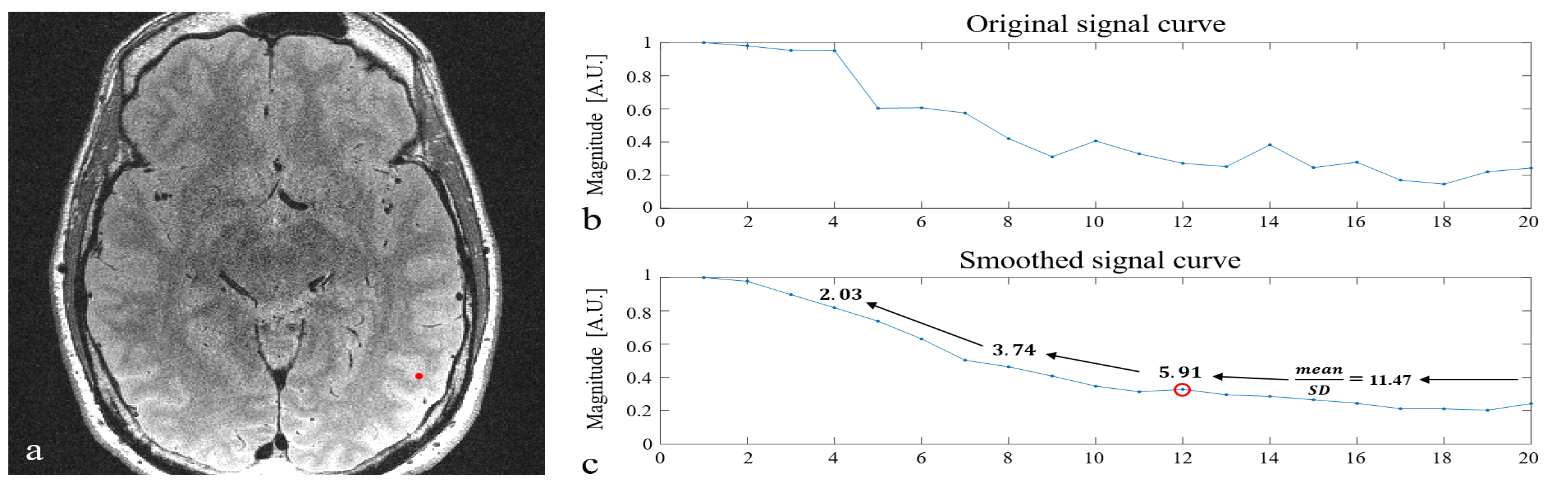


**Figure S1:** Example for the steps used by the MP-PCA denoising algorithm, in order to exclude noisy times points from the T_2_ fitting process. **(a)** Original pre-denoising image, corresponding to the second TE of a MESE acquisition. **(b)** The original signal decay curve of a single voxel, marked as a red dot in (a). **(c)** The signal decay curve after a 5-point moving average. For each group of values starting from the last echo, the inverse of the coefficient of variation (mean / SD) is calculated. The value of the inverse of the coefficient is initially high, containing only noisy data-points, and gradually decreases as the mean signal and SD increase. The point, at which this value drops below a threshold=6.0, is chosen as the last echo containing meaningful signal.

## II. Gibbs ringing artifacts is phantom scans

In this study, SNR was estimated by dividing the mean value within each sphere, with the SD of noise in the background ROIs. A second possible method for SNR estimation was to divide the mean value within each ROI with the SD of the signal within the same ROI. This method was abandoned due to Gibbs ringing artifacts, affecting the SD inside the spheres, and causing changes in the variability inside the spheres between different spatial resolutions.

**Figure S2:** Gibbs ringing artifacts shown on the first echo-time images of two MESE scans. Both scans were performed using the same FOV (160x160 mm^2^) and using matrix sizes of 256x256 (left) and 100x100 (right). As can be seen, both images, and particularly the low resolution one, are affected by Gibbs ringing artifacts, preventing reliable estimation of the noise pattern (i.e., the standard deviation of the signal) within each homogeneous sphere.

## III. Close up views of phantom spheres in original and denoised images

Fig. S3 presents a close-up view of the HPD phantom T_2_ values within spheres #3 and #4. The left panels show T_2_ values calculated from the original images, and the right panels show the corresponding T_2_ values calculated using denoised images. Color bars were separately adjusted to match the relevant range of T_2_ values within each sphere. A discretization of T_2_ values can be observed within the spheres, resulting from narrow dynamic range of the color bars and the discrete values retrieved from the EMC dictionary during fitting process. A clear decrease in the variability of T_2_ values resulted following the denoising process, as expected from a uniform phantom solution.

##
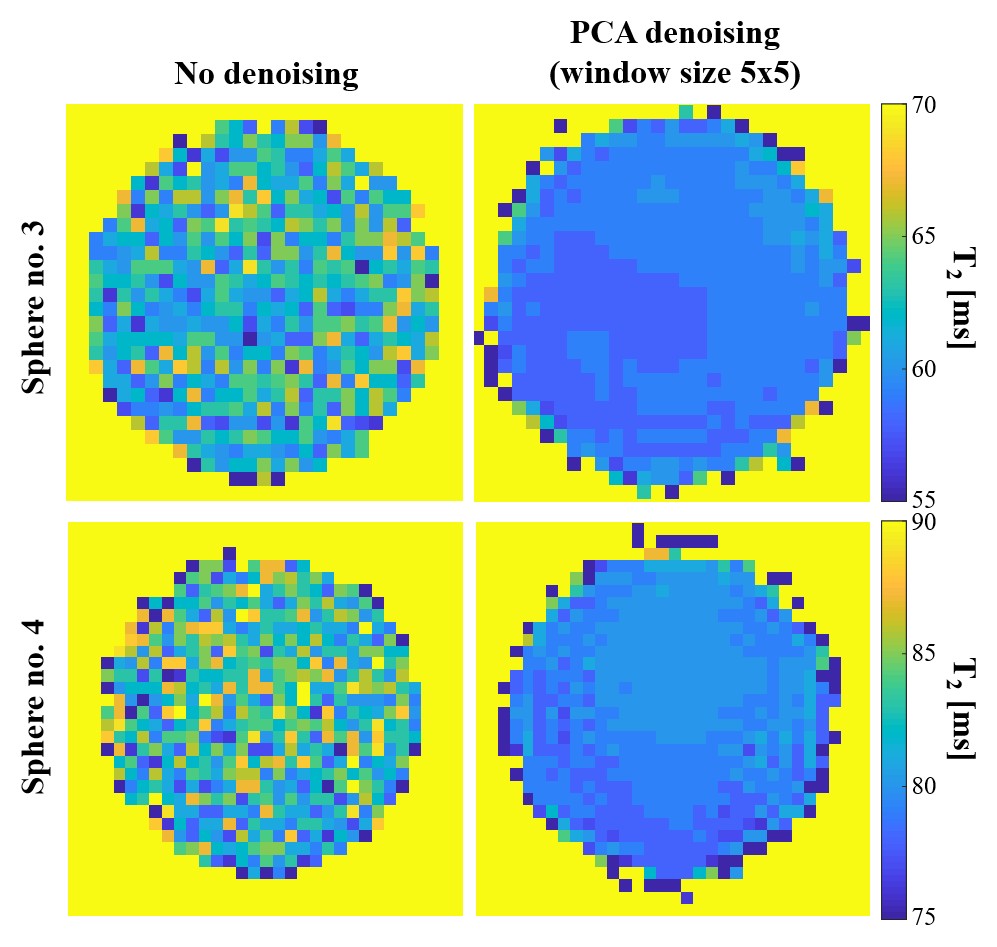


**Figure S3:** **Left column:** Close up view of the T_2_ maps constructed from original images for spheres 3 and 4 (top and bottom rows). **Right column:** Close up view of the T_2_ maps constructed from denoised images for the same spheres. Color axis was adjusted separately to the relevant T_2_ range of each sphere. A clear decrease in T_2_ values’ variability can be seen post-denoising.

## IV. Analysis of the level of noise removal across echoes

Further numerical analysis of the pattern of removed noise was performed on the phantom data. Noise images were calculated by subtracting the denoised images from the original images. The mean of the removed noise values was calculated per sphere and per echo time. Spheres characterized by lower T_2_ values showed an increase in noise mean, while spheres characterized by higher T_2_ showed a less significant trend. This can be explained by the noncentral chi distributed noise pattern induced by the use of magnitude images and the sum of squares method for combining images from different coils. As time progresses, the signal curve decreases in a rate that is determined by the T_2_ value of the sphere; a shorter T_2_ dictates a higher decay rate, implying lower SNR in the later echoes.


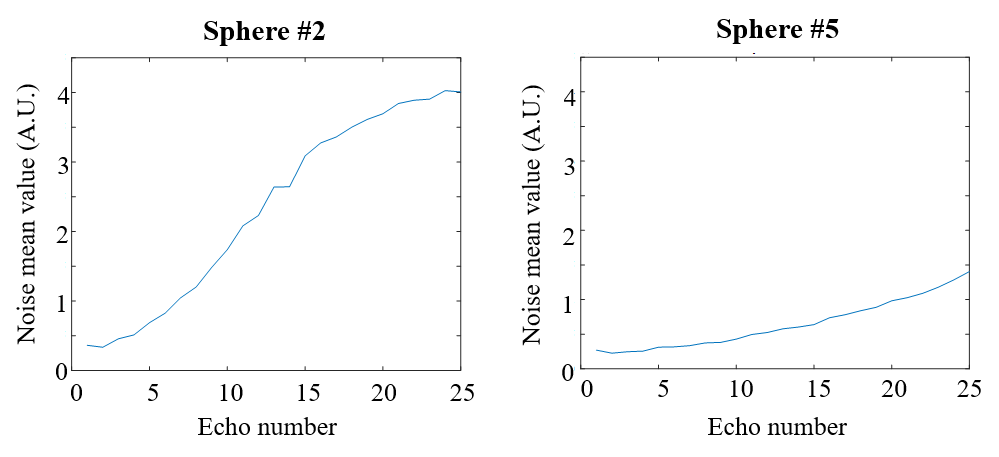


**Figure S4:** Mean of the removed noise across echoes, evaluated for internal ROIs of spheres #2 (~42 ms) and #5 (~120 ms) of the phantom. Noise was calculated by subtracting the denoised images from the original images. Analysis of sphere #2 revealed an increase in noise mean, while analysis for sphere #5 showed a less significant trend.

## V. Repeatability of the denoising algorithm

The stability of the MP-PCA denoising algorithm was tested by applying it on images acquired during three repeated MESE scans of the same anatomy. Two sets of T_2_ maps were generated from the original and denoised images. Fig. S4a-c presents original vs. denoised T_2_ maps corresponding to the three repeated scans. The mean value of each set of maps was also calculated and presented in Fig. 4d,h. Visual inspection shows high similarity between the denoised T_2_ maps.

The feature marked by the red rectangle was identified by an expert as an anatomical feature (a perivascular space). As can be seen in panels S4.e-g, this feature was preserved in the denoising process for all repetitions. The region marked by the white rectangle contains several voxels with exceptionally high T_2_ values. Comparing the spatial variance within this region (panels S4.a-c), it is likely that the variance within this region is affected by noise. The apparent noise removal in this region can be examined when comparing panels S4.a-c to panels S4.e-g.

**Figure S5:** T_2_ maps resulting from three identical MESE acquisitions of the same anatomy. **(a-c)** T_2_ maps generated from the original images; **(e-g)** T_2_ maps generated from denoised images; **(d)** and **(h)** show the averages of (a-c) and (e-g), respectively. The feature marked by the red rectangle was identified by an expert as an anatomical feature (a perivascular space); as can be seen in (e-g), this feature was preserved in the denoising process for all repetitions. The region marked by the white rectangle contains several voxels with exceptionally high T_2_ values. Comparing the spatial variance within this region in (a-c), it is likely that the variance of the presented T_2_ values is this region are affected by noise. The apparent noise removal in this region can be examined when comparing panels (a-c) to (e-f).

## VI. Spatial distribution of noise reduction

In order to obtain a visual illustration of the noise reduction, the difference between pre- and post-denoising was calculated for a sample pair of images. The difference map reflects a non-uniform spatial distribution of the noise being removed with some of the anatomical structures visible. Although we would not expect noise maps to exhibit structural information, this arises from fact that the algorithm chooses a different number of PCs in each window. For instance, the center of the anatomy is farthest away from the receive coils, thus having the lowest SNR. As can be seen in the relative difference map, this is the area that is most affected by the denoising.


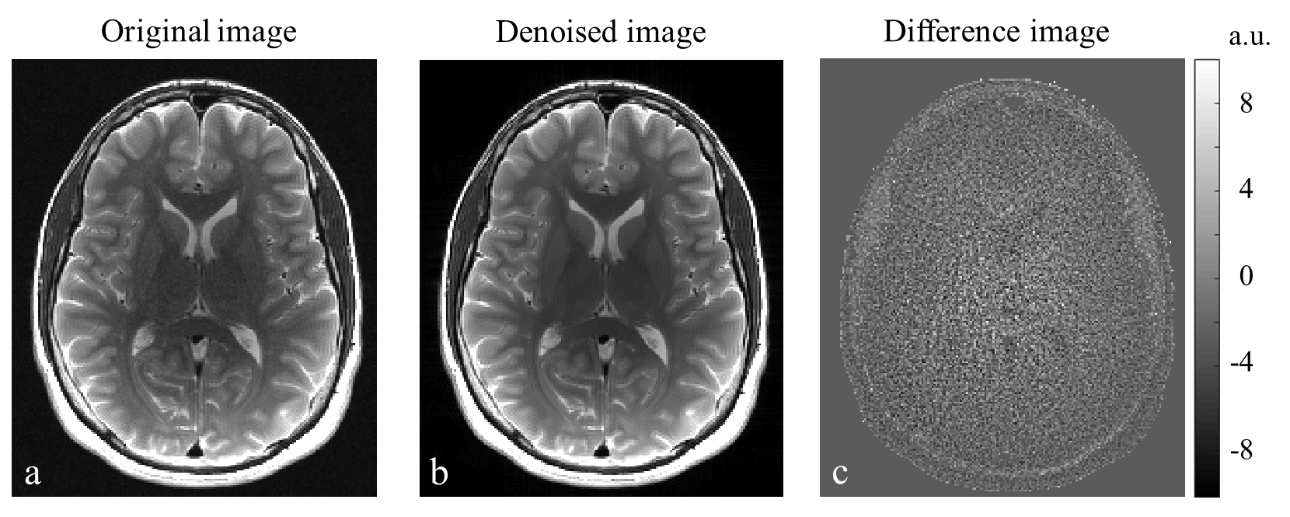


**Figure S6:** Spatial distribution pattern of removed noise **(a)** The original image acquired at the 8^th^ echo of an in vivo brain MESE scan. **(b)** The corresponding denoised image (window size 5x5x15). **(c)** Difference between the left and the middle images.

***VII. Feature preservation in in-vivo images***

Although edge preservation is visible in the previously shown simulations and phantom scans, they do not mimic anatomical features. We therefore demonstrate feature preservation in the in-vivo scans, examined by experts to ensure the preservation of anatomical features (a neuroradiologist with 11 years of experience and an orthopedic with 8 years of experience). **Fig. S7** below shows several zoom-in views into four selected images from two brain scans and a single knee scan.

******

**Figure S7:** Demonstration of feature preservation by comparing zoom-in views into four selected images. **(a-d)** First brain scan; **(e-f)** Second brain scan; **(g-h)** Knee scan. Left column: original T_2_ maps; Right column: denoised T_2_ maps.

***VIII. Window size selection for MP-PCA image denoising***

The window size for the MP-PCA image denoising process was selected empirically per scan. After testing different window sizes for different in vivo scans, we found that applying MP-PCA denoising using a too small window size resulted with “cloudiness” effects, while using a large window resulted in grainy images and poor denoising quality. This is demonstrated on denoised in-vivo images in Fig. S8. Further discussion on considerations involved with window size selection can be found in Veraart et al. (2016).

**Figure S8:** Examination of denoising results for brain imaging with different window sizes. Each row contains images of a different slice from the second high-resolution brain scan mentioned in the paper. Denoising with a small window size results with “cloudiness” effects, while using a large window results in grainy images.
